# Gestational Diabetes: Physical Activity Before Pregnancy and Its Influence on the Cardiovascular System

**DOI:** 10.3389/fped.2020.00465

**Published:** 2020-08-14

**Authors:** Christina Sitzberger, Renate Oberhoffer-Fritz, Kristina Meyle, Maike Wagner, Nadine Lienert, Oliver Graupner, Regina Ensenauer, Silvia M. Lobmaier, Annette Wacker-Gußmann

**Affiliations:** ^1^Faculty of Sports and Health Sciences Technische Univeristät Munich, Institute of Preventive Paediatric, Munich, Germany; ^2^German Heart Centre Department of Paediatric Cardiology and Congenital Heart Defects, Munich, Germany; ^3^Klinikum rechts der Isar, Department of Gynaecology and Obstetrics, Munich, Germany; ^4^Research Centre, Dr. von Hauner Children's Hospital, Ludwig-Maximilians-Universität München, Munich, Germany; ^5^Experimental Paediatrics, Department of General Paediatrics, Neonatology and Paediatric Cardiology, University Children's Hospital, Heinrich Heine University Düsseldorf, Düsseldorf, Germany

**Keywords:** gestational diabetes, physical activity, cardiovascular system, pregnancy, intima media thickness

## Abstract

**Objectives:** Gestational diabetes mellitus (GDM) is a common complication in pregnancy, affecting around 14% of all pregnancies each year. It will likely further increase, as obesity becomes more prevalent. The impact of GDM on cardiovascular changes in pregnant women and her child is still unclear. The aim of the study was to measure the effects of physical activity before pregnancy on the cardiovascular system in patients with GDM in pregnancy.

**Methods:** Two hundred and six pregnant women were included in this observational study. All participants were recruited at the tertiary level teaching University Hospital “Klinikum rechts der Isar” between 28 and 32 weeks gestation. Questionnaires dealing with pre-pregnancy daily and physical activity (PA) were evaluated. The cardiovascular status of the mothers included measurements of the intima-media thickness (IMT) of the carotid arteries. PA level was performed with a standardized 6-min-walking-test.

**Results:** Ninety-nine women with GDM with a mean age of 33.84 (± 4.7) years were examined. One hundred seven healthy pregnant women aged 32.6 (± 4.2) years served as controls. The mean weight in the study group was 73.0 (± 20.3) kg and 61.7 (± 9.5) kg in the control group. Based on the higher weight in the study group, the Body Mass Index (BMI) was also significantly higher than in the control group (26.3 ± 7.1 vs. 21.6 ± 3; *p* < 0.001). The frequency of PA was significantly higher in the control group (*p* < 0.001). The objective fitness level was worse in pregnant women with GDM compared to healthy controls (472 vs. 523 m, *p* < 0.001). PA before and during pregnancy was less performed in the study group (86 vs. 64.5%, *p* = 0.002; 69 vs. 45.7%, *p* = 0.003). Women who were physically inactive before pregnancy had a 3-times higher risk to develop GDM compared to active women (OR = 2.67). The IMT was significantly thicker in the study group (0.48 ± 0.042 mm vs. 0.45 ± 0.042) mm; *p* = 0.006).

**Conclusion:** Physical activity before pregnancy and a lower initial weight reduces the risk of developing GDM and cardiovascular risk factors in pregnancy. The development of prevention programs is certainly necessary.

## Introduction

The increasing wealth of industrial states in Europe leads to health problems such as obesity. In consequence, dyslipidemia, high blood pressure, and diabetes mellitus is much more common. In Europe, these diseases are responsible for ~1.9 million deaths per year (1). Due to the increasing affluence, the number of pregnant women with obesity, and gestational diabetes is also increasing rapidly. In addition, new and improved screening tools over the last years resulted in significantly more women being diagnosed with gestational diabetes (2–4).

Scientific studies, suggest that pregnant women with gestational diabetes have an increased risk of maternal and fetal morbidity during pregnancy and childbirth (5, 6). These women may also develop long-term risk conditions such as severe obesity and diabetes mellitus (type 2) (7, 8). Therefore, the number of cardiovascular sequelae such as disorders of the lipid metabolism, high blood pressure, myocardial infarction, stroke etc. in these mothers will increase rapidly in the future (4).

It is still unclear how and at which age gestational diabetes affects the child in the context of the so-called “fetal programming,” acquired to the first influence (9).

The “Barker hypothesis” postulates that “programming during embryonic and fetal life,” determines the set point of physiological and metabolic responses that carry into adulthood. Any stimulus at a critical period of embryonic and fetal development can result in developmental adaptations that produce permanent structural, physiological, and metabolic changes, predisposing an individual to cardiovascular, metabolic, and endocrine disease in adult life (10). Initial studies show that children after pregnancy with gestational diabetes can develop hypertension early on (11, 12).

A first starting point for primary prevention can be a simple and cost-effective method: physical exercise. The benefit of physical activity in healthy pregnant women has been demonstrated in several studies (13–15). However, in most of the studies, physical activity was only evaluated by questionnaires or only gave sports recommendations to the pregnant women and thus lacked a guided sports program (16–18). A few studies included a controlled aerobic exercise program and showed that it is associated with significant reduce of gestational diabetes (GDM) in overweight and obese pregnant women. These results are from a large prospective randomized clinical trial recruiting 300 singleton women with a mean pre-pregnancy body mass index of 26.8 kg/m^2^ (19). In contrast, the systematic review and meta-analysis by Malosso et al. showed a significant reduction of GDM with a controlled exercise programme in overweight and obese pregnant women (20). In contrast the study group of Poston et al. found, that mainly obese women were examined with regard to their risk of contracting GDM. No significant difference was found between the intervention group and the control group concerning dietary and activity-enhancing recommendations. With regard to the assumption that the year before pregnancy is the most important one for the development of GDM, obese women have an advance risk (21). However, these studies focused on obese women in general only and not on the cardiovascular outcome in particular. The systematic review by Shepard et al. also found a difference between the intervention group and the control group in the number of women suffering from GDM (15). Most studies in the review by Shepard et al., only gave recommendations for an active lifestyle for these women. Only in eight studies a controlled sports programme was provided to the women (15). In addition, to our knowledge there are no controlled studies that have systematically studied the effects of physical activity in patients with GDM on the maternal and infant cardiovascular system. Therefore, the aim of this study was to evaluate the influence of physical activity before pregnancy on the development of GDM and the risks to the cardiovascular system.

## Materials and Methods

Pregnant women with gestational diabetes were included in the prospective controlled observational study between August 2015 and December 2018. Healthy pregnant women served as controls. All participants were recruited at the tertiary level teaching University Hospital “Klinikum rechts der Isar” of the Technical University of Munich between 28 and 37 weeks gestation. Recruitment was carried out by the research team. The pregnant women, both for the study groups and the control group, were addressed during their routine examination. The suitability for this study, with regard to the inclusion and exclusion criteria, was checked in advance on the basis of the existing files. Gestational diabetes was diagnosed according to the German guidelines of AG-Diabetes and DGG 2003 (22). All women had a 75 g oral glucose tolerance test (oGTT). GDM was diagnosed if one criterion was found: fasting glucose >92 mg/dl, 1 h glucose >180 mg/dl and 2 h glucose level > 155 mg/dl.

Patients were recruited from the second trimester of pregnancy onwards, as gestational diabetes was mainly diagnosed beyond 24 weeks of gestational age. Inclusion criteria were full age and no pre-existing conditions of cardiovascular diseases. Exclusion criteria were additional cardiovascular or nephropathic diseases, multiple pregnancies, acute illness including infectious diseases, and premature labor.

The pregnant women were examined according to a predefined protocol. This included general health data (weight, height, BMI, oGTT, gestational age, gravida, para, blood pressure, food questionnaire) of the mother. The cardiovascular status of the mother included measurements of the intima-media thickness of the carotid arteries. Carotid scans were obtained with Aloka pro sound 6 Ultrasound by a trained operator. Left and right common carotids were examined in antero-lateral, postero-lateral directions. Only longitudinal images in which the intercases were very clear, were obtained (23). Four measurements in total were performed and the average was taken. Parameters of vascular stiffness were evaluated at the level of the common carotid artery just before bifurcation. The observation of the physical activity level of the mother before and during pregnancy was evaluated with a standardized questionnaire and a 6-min walking test. Activities were separated into four different groups: endurance sports, athletic sports, combined sports and light sports. Table 1 shows the classification. The individual sports were assigned with the corresponding metabolic equivalent (MET) according to the compendium of physical activities. The MET indicate how intense an activity is, in order to measure and classify the physical activity. A MET unit indicates how much oxygen the body uses at rest. *MET* = *3.5 ml O2/kg x min* or ~*1 kcal/kg x h*.

**Table 1:** Characteristics of the study (GDM) and control (healthy) group.

	**Study group (GDM)**			**Control group**			
	**No**	**Mean ± SD**	**Median**	**No**	**mean ± SD**	**median**	***P*-value**
Maternal age[years]	98	34.41 ± 4.64	34.23	107	32.96 ± 4.27	32.83	**0.021**
Maternal height [cm]	99	166.34 ± 6.19	167.00	107	167.75 ± 5.75	168.00	0.093
Maternal weight before pregnancy [kg]	98	72.75 ± 20.40	67.00	105	61.83 ± 9.47	59.00	**<0.001**
Body mass index before pregnancy [kg/m^2^]	96	26.24 ± 6.69	24.17	107	21.61 ± 4.28	21.09	**<0.001**
Weight gain during pregnancy [kg]	81	11.45 ± 6.85	11.60	89	15.30 ± 5.39	14.60	**<0.001**
Systolic blood pressure during pregnancy [mmHg]	88	111.82 ± 12.29		89	109.73 ± 11.5		0.574
Diastolic blood pressure during pregnancy [mmHg]	88	70.84 ± 10.94		89	68.26 ± 9.86		<0.103
Systolic blood pressure during pregnancy [mmHg] on release	81	117.91 ± 11.76		81	116.88 ± 16.69		<0.805
Diastolic blood pressure during pregnancy [mmHg] on release	81	76.1 ± 9.44		81	76.72 ± 9.65		<0.574
Gravida	88	2,34 ± 1,52	2,00	92	1,86 ± 0,97	2,00	**0.017**
Para	88	1,69 ± 0,98	1,00	92	1,46 ± 0,60	1,00	0.174
		**No. of patients**	**%**		**No. of patients**	**%**	
GDM in a previous pregnancy	^*^	32	^*^	^*^	4	^*^	^*^
Family history of diabetes	92	56	60,9	100	44	44,0	**0.019**
Insulin therapy	99	52	52,5				
Smoking	96	6	6,3	105	4	3,8	0.454

* *The questionnaire determined that GDM was present in a previous pregnancy, but not in how many pregnancies (in case of multiple pregnancies of a woman). Therefore, the percentage and the p-value cannot be calculated. The bold values indicate a significant difference*.

Through the MET's, the load tolerance, functionality and training capacity of individuals can be efficiently and consistently determined, regardless of their body composition. The classification of consumption is divided as follows: light: <3.0 METs, moderate: 3.0–6.0 METs, intensive: >6.0 METs (24).

The 6-min walking test was performed according to the guidelines of the American Thoracic Society (25). First a Polar® chest strap was attached to measure the maternal heart rate, then the patient rested for 10 min. The resting heart rate was quantified, and then the women began to walk. A walking round contained 90 m. All women were accompanied by an instructor and had to walk the round as often as they could within a time frame of 6 min. The heart rate was noted 3 and 6 min after beginning. The instructor stopped or told them to slow down if the heart rate was above a predefined limit. The limit was set with following equation: (220-age) × 0.7 (26). If the pregnant woman had any pain, shortage of breath or any sort of malaise, they paused while the time was running or—depending on severity—even stopped. Each pregnant woman was examined with a carotid scan and a physical activity test according to a pre-defined protocol. These examinations were done at the beginning of the study. The power analysis calculated a total sample size of *n* = 66 subjects.

### Statistical Analysis

The statistical analysis was done with SPSS version 25. Continuous variables were presented with mean ± standard deviation. For categorical variables of the study, numbers and percentages were determined. To evaluate the difference between the mean the independent samples *T*-test and Mann – Whitney – *U*-Test were performed. Pearson's chi-square test was used for categorical variables. Odds ratio was performed to indicate the strength of the relationship between developing GDM and physical activity. In a further multivariate logistic model, we analyzed the relationship of the parameters “age, family history of diabetes, physical activity, and BMI” on the development of gestational diabetes to find the strongest predictive parameter. A linear regression was performed to show the predictors for the changes in the IMT. The level of statistical significance was defined as *p* = 0.05. Subgroup analysis was done.

## Results

Overall 206 pregnant women were included in the study. 99 (47.8%) patients had gestational diabetes and 107 (52.2%) patients served as healthy controls. Fifty-two of all women with gestational diabetes were treated with insulin and 47 with dietary advices only. The mean gestational age at enrolment was 35.14 (SD ± 2.42) in the study group and 34.12 (SD ± 3.10) in the control group. The women in study group were more often pregnant and had more children than those of the control group (gravida: 2.34 ± 1.52; para: 1.69 ± 0.98 vs. gravida: 1.86 ± 0.97; para: 1.46 ± 0.60).

### Anthropometric Data of the Mother

The mean age of the two groups showed a difference of ~1.5 years (GDM = 34.31 ± 4.64 years, controls = 32.96 ± 4.64 years, *p* = 0.021). Before pregnancy, the study group had a significant higher weight (72.75 ± 20.40 vs. 61.83 ± 9.47 kg, *p* < 0.001) and a significant higher Body-Mass-Index (26.24 ± 6.69 vs. 21.61 ± 4.28, *p* < 0.001). None of the patients had arterial hypertension (RR> 140/90 mmHg) at the time of measurement. There was no significant difference in systolic (syst: 111.82 ± 12.29 mmHg study group vs. syst. 109.73 ± 11.52 mmHg control group) and diastolic blood pressure between the study and control group (syst: *p* = 0.224, dia: *p* = < 0.103). At release there was also found no significance in between the two groups (syst.: *p* = 0.805; dia: *p* = < 0.574). The weight gain during pregnancy in the study group was less than in the control group (GDM 11.45 ± 6.85 kg vs. control group 15.30 ± 5.39 kg). Thirty-two women in the study group and four women in the control group were diagnosed with gestational diabetes in a previous pregnancy. A positive family history of diabetes was found in more patients with GDM (60.9 vs. 44%, *p* = 0.019). Anthropometric data is summarized in Table 1.

### Vascular Diagnostics

The average IMT was significantly higher in the study group than in the control group (0.45 ± 0.42 vs. 0.48 ± 0.42; *p* =0.005). There was a significant correlation between BMI and IMT (ß = 0.00; *p* = 0.001; 95% CI: 0.00; 0.001). The general data is summarized with Table 2.

**Table 2:** Vascular diagnostic results in the study (GDM) and control (healthy woman) groups.

	**Study group (GDM)**			**Control group**			
	**No**	**Mean ± SD**	**Median**	**No**	**Mean ± SD**	**Median**	***P*-value**
Intima media thickness avg.(mm) total	96	0.48 ± 0.042	0.49	104	0.45 ± 0.042	0.46	**0.005**

*The bold values indicate a significant difference*.

### Blood Sugar Values

The mean fasting blood glucose value of the study group was 91.83 ±8.34 mg/dl. The mean values of 1 and 2 h oGTT were 168.61 ± 30.25 and 137.42 ± 32.77 mg/dl in the study group. The values of the control group were all within the normal range.

### Physical Activity: Six-Minutes Walking Test

Regarding the results of the 6-min walking test, the objective fitness level was lower in pregnant women with gestational diabetes compared to healthy controls. The resting heart rate was slightly higher in the study group (94 ± 17 vs. 91 ± 13 bpm, *p* = 0.178), just as the heart rate after 3 min (118 ± 14 bpm vs. 116 ± 13, *p* = 0.295). The heart rate after 6 min was equal in both groups (119 ± 14 bpm, *p* = 0.813). The completed distance was significantly lower in the study group (472 meters vs. 523 m, *p* < 0.001).

**Figure 1:**
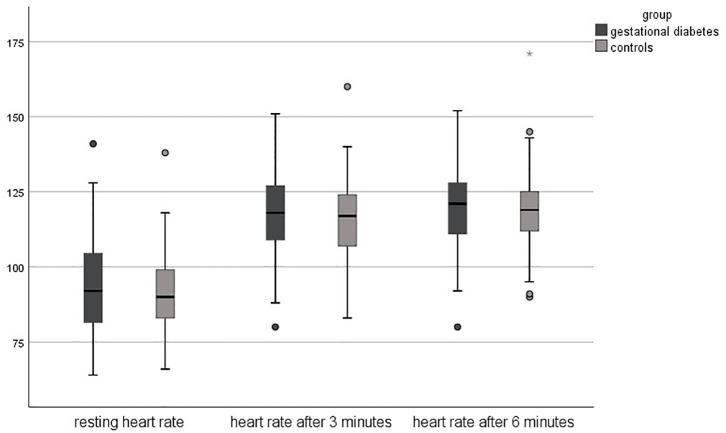
Heart rates during 6-minute walking test.

**Figure 2:**
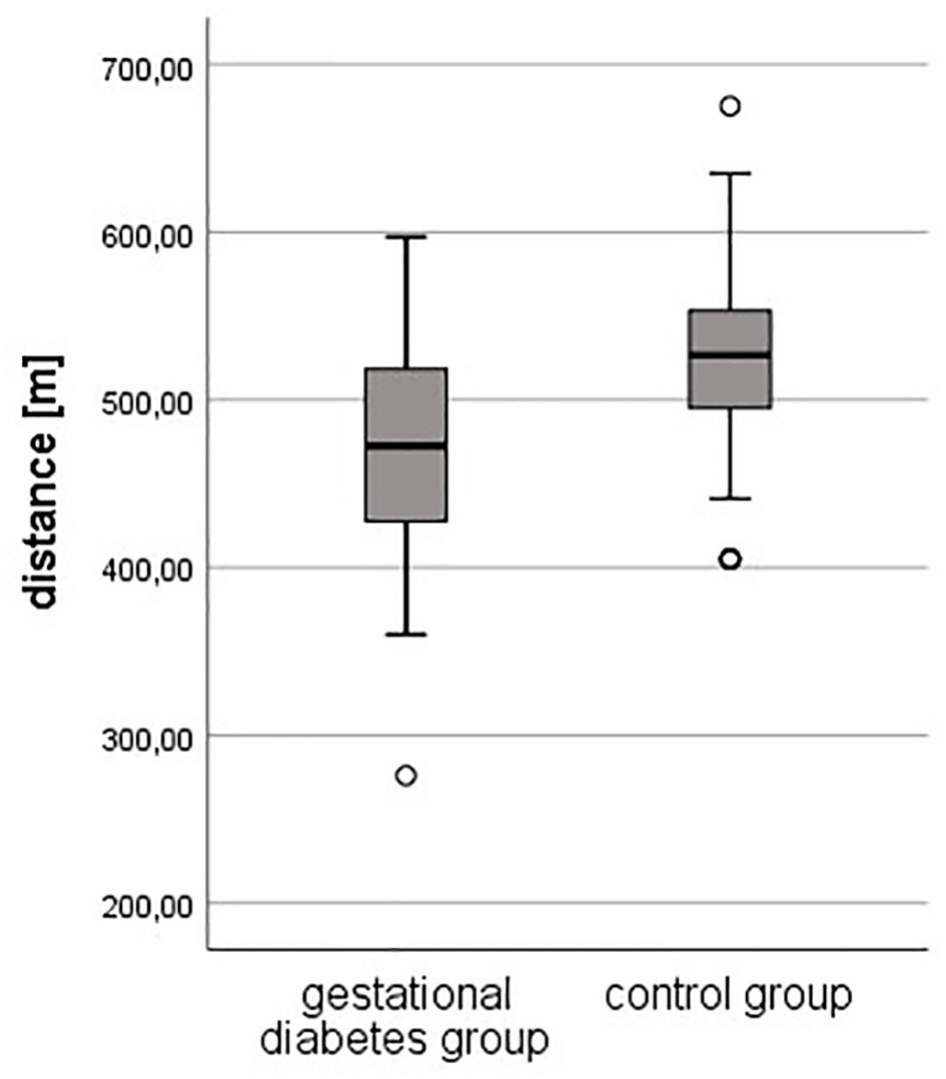
Completed distance during 6-minute walking test.

Figures 1, 2 shows the differences in the 6-min walking test.

### Physical Activity

**Figure 3:**
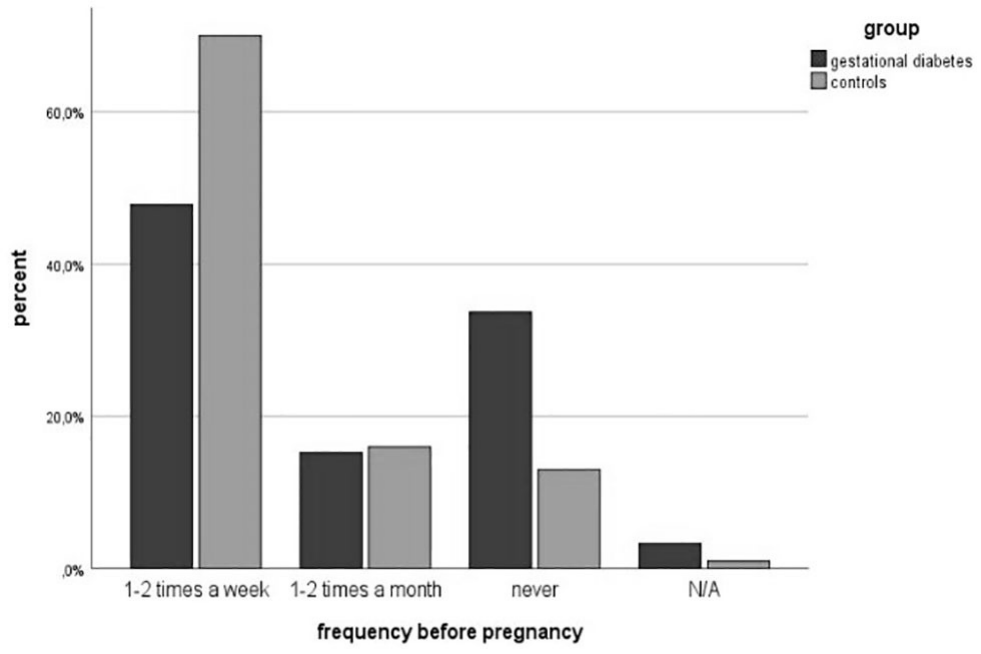
PA frequency before pregnancy.

**Figure 4:**
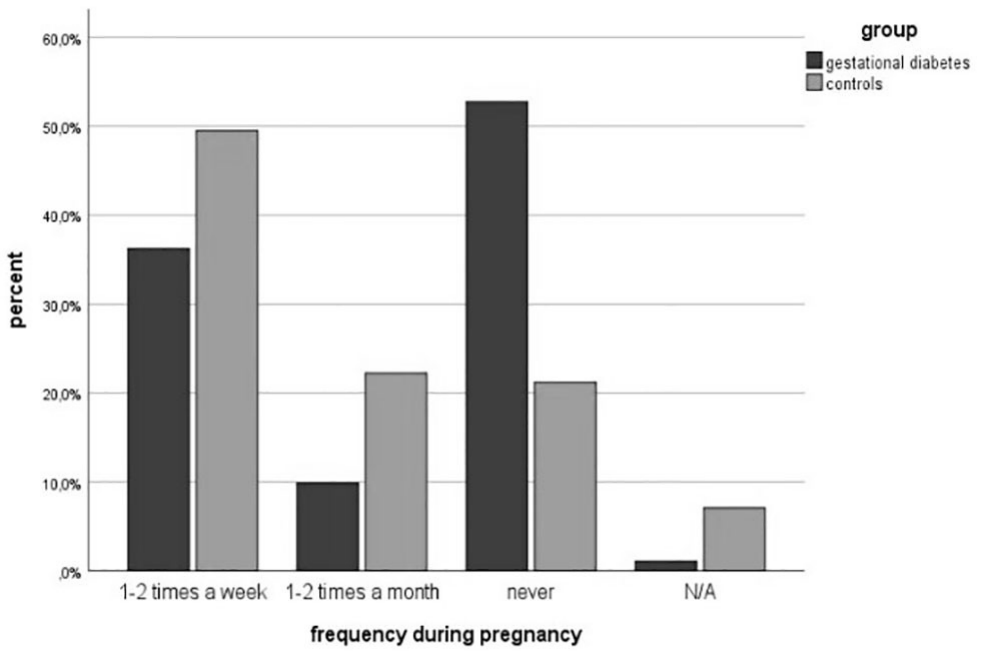
PA frequency during pregnancy.

Physical activity before pregnancy was performed less in the study group (64.5 vs. 86%, *p* = 0.002). The number of women with endurance sports was significantly lower in the GDM-Group (60.7 vs. 83.7%, *p* = 0.002). The number of patients participating in athletic (34.3 vs. 34.9%, *p* = 0.954) and combined sports (18.0 vs. 19.8%, *p* = 0.792) was equal. However, more women with gestational diabetes attended lighter sports (31.1 vs. 24.4%, *p* = 0.366). The classifications are summarized in Table 3. The frequency of doing physical activities was significantly lower in the study group (1-2x per week: 47.8 vs. 70%, 1-2x per month: 15.2 vs. 16%, *p* = 0.003). The average physical activity was divided according to the Compendium of Physical Activities (MET values) (27). The results showed a significant difference in MET values between both groups (3.34 ± 2.85 vs. 4.73 ± 2.55; *p* = 0.002). Figures 3, 4 show the different frequencies of physical activities before and during pregnancy.

**Table 3:** Classification of the different sports.

**Endurance**	**Athletic sports**	**Combined sports**	**Light sports**
Aerobic	EMS	Badminton	Aqua gymnastics
Endurance	Fitness	Ballet	Gymnastics
Speed ice-skating	defined back training	CrossFit	Pilates
Inline skating	Climbing	Figure skating	Back gym
Jogging		Soccer	Walking
Cross-country skiing		Handball	Yoga
Treadmill		Horseback riding	
Running		Skiing	
Biking		Squash	
Rowering		Dancing	
Swimming		Table tennis	
Spinning		Volleyball	
Hiking		Wakeboard	
Zumba			

Physical activities were less performed during pregnancy in the study group (46.2 vs. 69.0%, *p* = 0.003). The number of patients doing endurance sports decreased in the study group from 60.7% before pregnancy to 43.2% during pregnancy and in the control group from 83.7% before pregnancy to 63.4% during pregnancy. In athletic (15.9 vs. 18.3%, *p* = 0.741), combined (6.8 vs. 8.5%, *p* = 0.751) and light sports (54.5 vs. 56.3%, *p* = 0.851), the differences were slight within the two groups. The frequency of doing sports was comparable to the frequency before pregnancy. The gestational diabetes group did less exercise (1-2x a week: 36.3 vs. 49.5%, 1-2x a month: 9.9 vs. 22.2%, *p* < 0.001). This also corresponds to less daily activity in the study group (walking 53 vs. 69 min per day, *p* = 0.144). Before pregnancy 58 patients were active and 41 did not do any physical activity at all in the study group, whereas 85 were active in the healthy control group. Comparing the activity level before pregnancy to the development of GDM in pregnancy, the results show that inactive women have a three times higher risk to develop GDM compared to active women (OR = 2.67) The result was statistically significant (*p* = 0.02). The multivariate logistic model indicated the well-known significant impact of the BMI (ß = 0.17; *p* < 0.00; 95% CI 1.09; 1.30 OR = 1.19) and age (ß=0.11; *p* = 0.03;95%CI 1.04; 1.21;OR = 1.21) on the development of gestational diabetes. We further found that PA before pregnancy has a significant influence on the development of gestational diabetes (ß = −1.14; *p* = 0.04; 95%CI 0.14; 0. 69; OR = 0.31). The family history of diabetes didn't show any significance (ß = 0.16; *p* = 0.62; 95%CI 0.59; 2.34; OR = 1.18).

### Subgroup Analysis of the Physical Activity Level

Subgroup analysis of the gestational diabetes group was made by separating the group into two different physical activity levels (above and below average) concerning the 6-min walking test (distance 472 vs. 523 m, *p* < 0.001).

Concerning treatment strategies woman with an above-average physical activity had less insulin (41.2 vs. 57.9%, *p* = 0.157) and more dietary advices (58.8 vs. 42.1%).

The group with above-average physical activity level resulted in a lower birth weight (3,266 ± 324 vs. 3,449 ± 398 g, *p* = 0.056) compared to those with a below average activity level in the same group. The birth percentile was significantly lower in the above-average group (36.6 ± 22.0 vs. 52.4 ± 25.4, *p* = 0.013).

## Discussion

The main point of this study was that there is a link between physical activity in the year before pregnancy and the onset of gestational diabetes. Women who were physically inactive before pregnancy had a nearly 3-times higher risk to develop GDM compared to active women (OR = 2.67). We also found that the intima media thickness of the carotid arteries in women with GDM was significantly higher. Almost half of the women were diagnosed with GDM in an earlier pregnancy. The negative influence of GDM could indicate impaired vascular health. However, further controlled studies are needed to assess the vascular status before pregnancy.

The overall benefit of physical activity in healthy pregnant women has been demonstrated in several studies (27, 28). For example, Kihlstrand et al. proofed a positive effect of aqua gym during pregnancy on the occurrence of back pain (29). Kagan and Kuhn, showed that suitable sports for women during pregnancy and after birth are aerobic endurance sports with the components running, walking and rhythmic movements, as well as the stabilization of certain body positions, especially under the stress of the major muscle groups (30). Guelfi et al. demonstrated also the significant effect of maternal fitness between a 14 weeks supervised home—based exercise intervention vs. standard care on cardiovascular risk factors (31). Dye et al. found similar results (32). Our study agree with the already existing literature and also shows that physical activity has a positive influence on pregnancy.

Further it is stated that women who are active before pregnancy have a reduced risk of developing GDM, although cardiovascular changes were not examined in detail (16). A review by Russo et al. for example showed that physical activity can reduce the risk of developing GDM up to 28%. Sport reduces the risk of getting GDM even unaffected of other health factors (33). A higher physical activity level before and during early pregnancy shows a lower prevalence of GDM, which was also evident in our study. Women with GDM do less or no exercise per week. Our study showed that women with GDM achieved lower METs per week, and that these women had a lower level of fitness in an objective fitness test: the 6-min run. Zavorsky et al. showed that it is necessary to have at least 16 Mets hours per week to reduce the risk of GDM (34). The systematic review and meta-analysis by Davenport et al. found that being physically active for at least 600 Mets minutes per week to reduce the possibility of developing GDM by 25% (35). Therefore, it might be possible and useful to motivate pregnant women, based on the METs per week, obtained from data of our study and others. Sports with METs of 3 (moderate intensity) or more (vigorous intensity) according to the Compendium of physical activities might be: Exercise at least 5 times a week for at least 30 min before pregnancy to reduce the risk of gestational diabetes in pregnancy (24). In summary it depends on how long a sport is performed and not on which type of physical activity. However, controlled studies are further needed to re-evaluate these results. The intima media thickness of the A. carotis and the change of arterial stiffness might be a useful tool to monitor women's cardiovascular health.

It has to be considered, that women with GDM have a high risk of hypertensive disorders, which in turn is also associated with increased maternal arterial stiffness (36). In addition, the mechanisms that could increase arterial stiffness and IMT are complex. These include, for example, the arterial remodeling, oxidative stressor, and endothelial dysfunctional (37). Nevertheless, in this study, the results of the cardiovascular examinations showed that women with GDM had a significantly higher intima media thickness. None of the patients had arterial hypertension history or hypertension at the time of measurements. Women with gestational diabetes tend to be older and have a higher body mass index (BMI) than healthy women. The multivariate logistic model indicated the well-known significant impact of the BMI and age on the development of gestational diabetes. We further found that PA before pregnancy has a significant influence on the development of gestational diabetes. Women with a higher BMI are usually, physically less active and tend to have a higher BMI. To reduce the risk of developing gestational diabetes, physical activity before pregnancy could be an additional valuable tool to improve the health of the mother. Due to the close supervision of pregnant women with GDM by nutritionists and diabetologists the weight gain during pregnancy in the study group was less than in the control group.

A clear understanding of the relationship between GDM and cardiovascular changes is important and must have an impact on future research, lifestyle and health risks, as gestational diabetes is already the most common metabolic disorder in pregnancy. The frequency of the metabolic syndrome and its sequelae will increase significantly and make our already aging society even more morbid. The question increasingly arises of how to counteract this rapid development of health problems. Our study shows that physical activity before pregnancy could improve the health of these women.

### Limitations

There were some limitations in this study. First, it was just an observational study and not an interventional study. The physical activity of the participants was only surveyed and could not be checked for obvious correctness. The pregnant women were recruited in the second trimester in pregnancy as part of their check-up at the obstetrical department. Therefore, no specific cardiovascular data could be obtained before pregnancy. Thus, no conclusions can be drawn on the physical activity and the cardiovascular parameters such PWV and IMT before the study.

## Conclusion

Physical activity could be a very helpful tool to improve health problems in pregnancy. The activity should be measured by an objective fitness tests, and cardiovascular changes should be ideally measured before, in and after pregnancy. We found that the intima media thickness might be a useful tool to monitor cardiovascular changes for women with gestational diabetes. However, further controlled studies are needed to prove these results. Counseling parents and long-term care for the health of both, the mother and her child, should be improved and prevention strategies including controlled fitness programs should be developed.

## Data Availability Statement

The datasets generated for this study are available on request to the corresponding author.

## Ethics Statement

The studies involving human participants were reviewed and approved by Ethics Committee of the School of Medicine of the Technical University of Munich (464/15s). The patients/participants provided their written informed consent to participate in this study. Written informed consent was obtained from the individual(s) for the publication of any potentially identifiable images or data included in this article.

## Author Contributions

CS and AW-G study concept and design, analysis and interpretation, drafting of the manuscript, and final approval of the version to be published. RO-F supervision, and final approval of the version to be published and agreement with all aspects of the work. KM, MW, NL, OG, RE, and SL substantial contributions to data acquisition. All authors critically revised the manuscript and approved it for publication.

## Conflict of Interest

The authors declare that the research was conducted in the absence of any commercial or financial relationships that could be construed as a potential conflict of interest.
